# Silver-modified porous polystyrene sulfonate derived from Pickering high internal phase emulsions for capturing lithium-ion

**DOI:** 10.1039/c8ra09740b

**Published:** 2019-03-05

**Authors:** Xiaojing Wang, Xueping Chen, Yinxian Peng, Jianming Pan

**Affiliations:** School of Environmental and Chemical Engineering, Jiangsu University of Science and Technology Zhenjiang Jiangsu 212003 China pyxhx@just.edu.cn +86 88791800 +86 88791708; School of Chemistry and Chemical Engineering, Jiangsu University Zhenjiang 212013 China pjm@ujs.edu.cn

## Abstract

Adsorption separation based on porous polystyrene sulfonate is an important method of extracting lithium ion (Li^+^). In this work, silver-modified porous polystyrene sulfonate (PHIPEs-SS-Ag) derived from Pickering high internal phase emulsions was fabricated for the selective binding of Li^+^. PHIPEs-SS-Ag possessed porous polymer matrix, sufficient sulfonic acid functional groups, and uniformly immobilized silver particles, which were beneficial for improving mass transfer, binding amount and antifouling performance. In batch mode experiments, the adsorption capacity reached a maximum value (*i.e.* 14.09 mg g^−1^) under alkaline conditions, and the adsorption mechanism between PHIPEs-SS-Ag and Li^+^ was electrostatic attraction. PHIPEs-SS-Ag exhibited fast binding kinetics at 25 °C (*i.e.* 300 min), and the maximum monolayer adsorption amount from the Langmuir model for Li^+^ are 59.85 mg g^−1^, 35.06 mg g^−1^, and 27.09 mg g^−1^ at 15 °C, 25 °C, and 35 °C, respectively. Moreover, PHIPEs-SS-Ag displayed excellent selectivity for Li^+^ in the presence of K^+^, Mg^2+^, and Na^+^, and maintained 80.71% of the initial adsorption capacity after seven sequential cycles of adsorption–regeneration. Therefore, this work opened up a universal route for the development of composite adsorbents for the specific separation of Li^+^.

## Abstract 2

Adsorption separation based on porous polystyrene sulfonate is an important method of extracting lithium ion (Li^+^).

## Graphical abstract

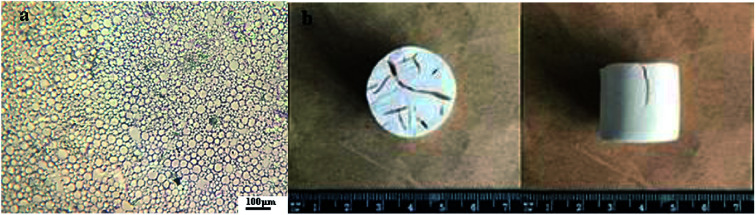

## 1. Introduction

Lithium is widely used in various fields of national economy such as ceramics, glass, lithium-ion batteries, and nuclear energy due to its excellent properties.^1^ The demand for lithium increases year by year, with the continuous expansion of the application field.^2,3^ Therefore, it is necessary to improve the production methods of lithium ions in order to meet the huge market demand. Lithium-rich minerals and salt lakes are the two major sources of lithium.^4,5^ China has a very rich resource of salt lakes, with a lithium content as high as 25%.^5^ The extraction of lithium ion from salt lakes has the advantages of being low price and environmentally friendly. However, current extraction techniques still have many difficulties, especially because of the presence of interfering ions including K^+^, Na^+^, and Mg^2+^.^6,7^ Until now, enormous methods such as calcination, precipitation and solvent extraction have already been used to improve the extraction of lithium.^8^ Unfortunately, the calcination method causes serious corrosion to the equipment, which may cause environmental pollution.^9^ Moreover, the high price of the precipitant used in the precipitation method leads to the poor prospect of industrial application.^10^ Furthermore, a large amount of organic waste liquid is easily produced by solvent extraction.^11^

A fantastic technique available is called adsorption separation,^12–14^ which shows promise in this regard due to the advantages of its low cost, high efficiency, and easy processing and recycling ability. It has been successfully used for gas separation, air purification, and wastewater treatment.^15^ S. Choudhury^16^*et al.* reported a lithium-adsorption material with low efficiency due to the long mass diffusion path length. High internal phase emulsions (HIPEs), in which the internal phase volume fraction exceeds 74%, are the novel templates for the fabrication of porous polymers.^17–19^ In the case of using colloidal particles as a stabilizer or colloidal particles, the formed emulsions are referred to as Pickering HIPEs.^20,21^ Porous polymers derived from HIPEs are potential adsorbents because they are capable of improving mass transfer and the efficiency of adsorption. In addition, the introduction of stabilizer particles could enhance mechanical performance. For example, a porous material with a highly interconnected pore network derived from a Pickering HIPEs template was reported to effectively bind copper ions.^22^

Recently, Chen's group proposed an important work about metal–organic frameworks (MOFs) modified with sulfonic acid groups for the extraction of lithium ions.^23^ In this work, sulfonic acid groups were considered as the available binding agent for lithium ions. However, the introduction of sulfonic acid groups into porous material *via* HIPEs has not been reported. To date, biofouling, the process by which organisms attach to underwater adsorbents, poses a major economic burden for separation industries. Fouling organisms that settle on submerged surfaces increase hydrodynamic drag, lower binding sites, and, in turn, increase non-specific adsorption.^24,25^ Silver is an effective antibacterial agent, which has been immobilized on different support materials for a wide range of antimicrobial and antifouling performance applications.^26^ To the best of our knowledge, using Pickering HIPEs to produce the nano-silver immobilized porous material was rarely reported.

In this work, halloysite nanotubes (HNTs),^27^ a very promising nanomaterial because of its hollow tubular morphology, large specific area, and tunable surface chemistry, was first considered as a stabilizer to form oil-in-water O/W Pickering HIPEs. Then, porous polystyrene sulfonate derived from Pickering HIPEs (PHIPEs-SS) was simply prepared, and sodium *p*-styrene sulfonate (SS) in the external phase was adopted as the functional monomer. Finally, silver-modified porous polystyrene sulfonate (PHIPEs-SS-Ag) was fabricated *via* an *in situ* approach in the presence of NaBH_4_ solution. The adsorption kinetics, equilibrium, selectivity and regeneration of PHIPEs-SS-Ag towards lithium ions were thus investigated, and the antimicrobial performance was also discussed.

## 2. Experimental

### 2.1 Materials

Halloysite nanotubes (HNTs) were obtained from Zhengzhou Jinyangguang Chinaware Co., Ltd. (Henan, China). Tween-80, acrylamide (AM), *N*,*N*′-methylenebisacrylamide (BAM, 99%), sodium *p*-styrene sulfonate (SS, 90%), and silver nitrate (AgNO_3_, 99.0%) were supplied by Aladdin Reagent Co., Ltd. (Shanghai, China). Ammonium persulfate (APS, 98%), liquid paraffin, LiCl·H_2_O, ethanol, acetone, sodium chloride (NaCl), and sodium borohydride (NaBH_4_) were purchased from Sinopharm Chemical Reagent Co., Ltd. (Shanghai, China). In addition, peptone, yeast extract, and agar were purchased from Sigma-Aldrich (Germany). *E. coli* (ACCN, KJ880039) strains were used for antibacterial experiments. The deionized water used in the experiment was filtered with an ultrapure water instrument, and all chemicals were used without further purification.

### 2.2 Characterization

The images of the Pickering HIPES droplets were obtained *via* optical microscopy (OM, Shanghai Peter EM (BM) Optical Instrument Manufacturing Company Limited, China). The morphologies of PHIPEs-SS and PHIPEs-SS-Ag were observed by field emission scanning electron microscopy (SEM, JSM-7100F; JEOL Ltd., Japan). Energy dispersive spectrometry (EDS) data were examined through an SEM instrument. Infrared spectra (4000–400 cm^−1^) were performed based on Fourier transform infrared spectrometry (FTIR, NEXUS-470; Nicolet, USA). The curves of the TGA analysis were taken by an integrated thermal analyzer (TGA, Perkin-Elmer, U.S.A.) under a nitrogen atmosphere ranging from 25 °C to 800 °C at a heating rate of 5.0 °C min^−1^. The specific surface area was determined by the Brunauer–Emmett–Teller method (BET, ASAP 2420 HD88, Micromeritics, U.S.A.). The elemental analysis of two polymer materials was detected *via* an element analyzer (EL, FLASH112A; Thermo Electron Ltd., American). XPS spectra of PHIPEs-SS and PHIPEs-SS-Ag were recorded through X-ray photoelectron spectroscopy (XPS, ESCALAB 250; Thermo Electron Ltd., American). The concentration of lithium ions was measured by inductively coupled plasma emission spectrometry (ICP, VISTA-MPX; Varian Australia Ltd., Australia).

### 2.3 Preparation of porous polystyrene sulfonate derived from Pickering HIPEs (PHIPEs-SS)

**Scheme 1:**
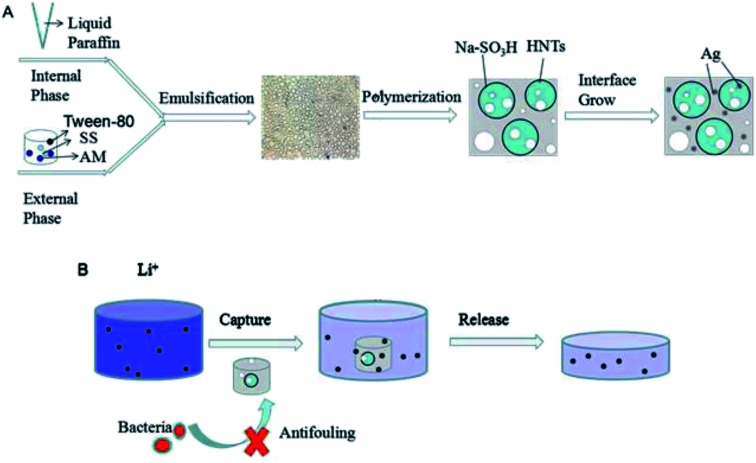
Schematic for the preparation procedure of PHIPEs-SS-Ag (A) and its adsorptive behavior towards Li^+^ (B).

**Fig. 1:**
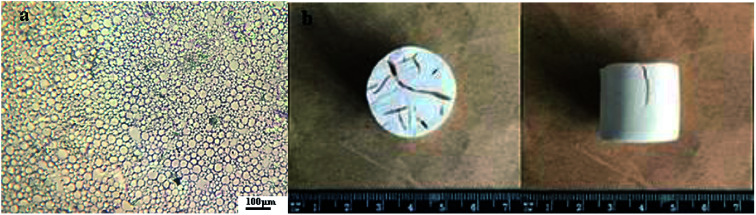
Micrograph of the Pickering O/W HIPEs-SS (a); PHIPEs-SS monolith (b).

The fabrication of PHIPEs-SS was carried out by an emulsion-templated method. Firstly, a uniform aqueous phase was prepared by dispersing 0.08 g of HNTs and 0.04 g of Tween-80 into 4.0 mL of deionized water, after which the mixture was sonicated for 30 min. Then, 0.7 g of AM, 2.5 g of SS, 0.309 g of BAM, and 0.02 g of APS were continuously injected to the aqueous phase (*i.e.* external phase), and this mixture was continuously stirred for 1.0 h (Scheme 1A). To obtain stabilized O/W Pickering HIPEs, 16 mL of liquid paraffin as the oil phase (*i.e.* internal phase) was added into the mixed aqueous phase dropwise under continuous stirring for 3.0 h. Next, the as-prepared emulsion was moved to a 25 mL ampere bottle and polymerized at 70 °C for 24 h. Afterward, the product (*i.e.* PHIPEs-SS) was oven-dried at 50 °C (Fig. 1) overnight in a vacuum oven until it reached a steady weight. Finally, PHIPEs-SS as washed with acetone *via* Soxhlet extraction to extract the internal oil phase and small molecules. In order to obtain the optimized amount of SS, 2.5 g (0.012 mol) and 3.0 g (0.015 mol) of SS were used to prepare PHIPEs-SS, and the products were named as PHIPEs-0.012SS and PHIPEs-0.015SS, respectively.

### 2.4 Synthesis of silver-modified porous polystyrene sulfonate (PHIPEs-SS-Ag)

An *in situ* approach was used to fabricate PHIPEs-SS-Ag. 10 mg of PHIPEs-SS was soaked in a centrifuge tube containing 10 mL of AgNO_3_ (1.36 mg, 8.0 μmol) solution. Subsequently, the centrifuge tube was immediately sealed after nitrogen purging and was then shaken gently. After 24 h, the soaked foams were taken out and placed directly into boiling aqueous NaBH_4_ solution (24.2 mg, 640 μmol, 20 mL) for 15 min (Scheme 1A). Finally, silver-modified porous polystyrene sulfonate (PHIPEs-SS-Ag) was washed with deionized H_2_O and dried in a vacuum oven at about 70 °C.^24^ The products using PHIPEs-0.012SS and PHIPEs-0.015SS were named as PHIPEs-0.012SS-Ag and PHIPEs-0.015SS-Ag, respectively.

### 2.5 Antimicrobial activity

The antimicrobial activity of PHIPEs-SS and PHIPEs-SS-Ag were detected against the model *E. coli* bacterial strains by forming a zone of inhibition. The LB (Luria–Bertani) broth^28^ contained 1.0 g of peptone, 0.5 g of yeast extract, 0.5 g of sodium chloride, 3.0 g of agar, and 100 mL of water. 40–50 μL of bacteria was added to 4.0 mL of the nutrient solution above, and the bacteria were cultured by shaking for 15–20 h. Then, 1.0 mL of diluted bacteria and 20 mL of nutrient solution were transferred into the Petri dish, and the mixture was shaken evenly until the liquid showed micro-coagulation. When the cell number reached 10^8^ CFU, a piece of filter paper (2.0 cm), which was soaked in the material (*i.e.* PHIPEs-SS and PHIPEs-SS-Ag) dispersion (10 mg mL^−1^), was placed onto the Petri dish, and this plate was allowed to grow for 24 h at 37 °C.^29^ All pharmaceutical tools were sterilized by autoclave and operated at the beginning of a sterile bench near the flame.

### 2.6 Static adsorption experiments

In the static adsorption experiments, adsorption kinetics and the equilibrium isotherm were considered, and factors such as contact time and the initial concentration of Li^+^ upon adsorption were studied. In the kinetic experiments, PHIPEs-SS-Ag (10 mg) was placed in a 10 mL centrifuge tube in the presence of 10 mL of Li^+^ solution with an initial concentration of 150 mg L^−1^ in a water bath (298 K). After the desired contact time (10 min to 720 min), PHIPEs-SS-Ag was collected by high-speed centrifugation, and the Li^+^ concentration in the supernatant was detected by inductively coupled plasma emission spectrometry (ICP-ES).^30^ The number of adsorbates adsorbed at time *t* (*Q*_*t*_, mg g^−1^) was calculated using the following equation, eqn (1):1
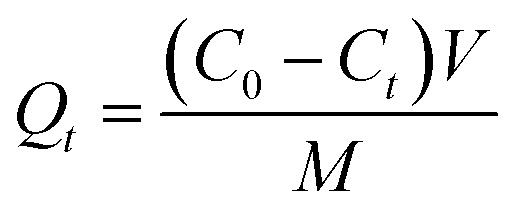
where *C*_0_ (mg L^−1^) represented the initial concentrations of Li^+^, *C*_*t*_ (mg L^−1^) is defined as the residual concentration in the solution at time *t*, *C*_e_ (mg L^−1^) is the equilibrium concentrations of adsorbates in the solution, *V* is the volume of the solution (mL), and *M* is the mass of PHIPEs-SS-Ag (mg).

To study the adsorption isotherm, a range of initial concentrations (50 mg L^−1^ to 400 mg L^−1^) of Li^+^ were mixed with 10 mg of PHIPEs-SS-Ag in centrifuge tubes at 288 K, 298 K and 308 K, respectively. After incubation for 6.0 h, the amount of Li^+^ in the filtrate was determined by ICP-ES. The equilibrium binding amount *Q*_e_ (mg g^−1^) was also calculated by eqn (1), but *C*_*t*_ was replaced by the equilibrium concentration (*C*_e_, mg L^−1^).

### 2.7 Binding specificity and regeneration experiments

In order to measure the specificity of PHIPEs-SS-Ag, K^+^, Mg^2+^, and Na^+^ were selected to be compared with Li^+^ under the same batch conditions. In specific binding experiments, 10 mg of PHIPEs-SS-Ag was added to 10 mL of single adsorbate solutions of Li^+^, K^+^, Mg^2+^, and Na^+^ (25 mg L^−1^) at 25 °C, respectively. After incubating for 6.0 h, PHIPEs-SS-Ag was collected by centrifugation, and the concentrations of Li^+^, K^+^, Mg^2+^, and Na^+^ (ref. 31) in the filtrate were detected by ICP-ES.

In regeneration experiments, the binding amounts of PHIPEs-SS-Ag were analyzed after seven adsorption–desorption cycles. PHIPEs-SS-Ag was firstly dispersed in Li^+^ solution with a concentration of 150 mg L^−1^ for 6.0 h at 25 °C, after which the binding amount was calculated. Then, the collected samples were washed with 0.5 M hydrochloric acid to remove captured Li^+^.

## 3. Results and discussion

### 3.1 Preparation of Pickering O/W HIPEs

The preparation procedure for PHIPEs-SS-Ag was illustrated in Scheme 1A. Firstly, Pickering O/W HIPEs with an internal-phase volume fraction of 80% were formed adopting HNTs and few Tween-80 as the emulsifier, in which the aqueous phase was the external phase and the oil phase was the internal phase. The water phase consisted of AM, SS, BAM, APS, and 4.0 mL of H_2_O, and the oil phase contained 16 mL of liquid paraffin. Secondly, after curing at high temperature, light PHIPEs-SS foams were fabricated.^32^ Finally, silver nanoparticles were modified onto the surface of macroporous monolith *via* the reaction between immobilized Ag^+^ and NaBH_4_.^33,34^ As-prepared PHIPEs-SS-Ag possessed the merits of porous structure and abundant sulfonic acid groups, which were beneficial for the rapid and effective capture of Li^+^.

### 3.2 Characterizations of PHIPEs-SS-Ag

Fig. 1 showed the images of the Pickering HIPEs-SS droplets (a) and Pickering HIPEs-SS (b). Fig. 1a showed that the emulsion droplets were uniform without obvious demulsification (the droplet microscope images taken after 12 hours of placement), which is sufficient to prove the stability of the emulsion. The substance Pickering HIPEs-SS before curing has a high viscosity, but after curing, it possesses a light foam column structure, as shown in Fig. 1b.

**Fig. 2:**
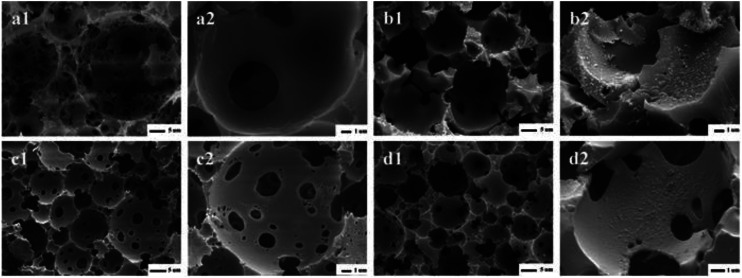
SEM images of PHIPEs-0.012SS (a1 and a2), PHIPEs-0.012SS-Ag (b1 and b2), PHIPEs-0.015SS (c1) and Area (c2), PHIPEs-0.015SS-Ag (d1) and Area (d2), respectively.

Fig. 2 showed the SEM images of PHIPEs-0.012SS (a1 and a2), PHIPEs-0.012SS-Ag (b1 and b2), PHIPEs-0.015SS (c1 and c2), and PHIPEs-0.015SS-Ag (d1 and d2), respectively. Four samples all possessed an open-cell structure with interconnecting pores as expected, and the high porosity endowed available permeability and low density.^35^ PHIPEs-0.015SS had many more interconnecting pores and open-cells than those of PHIPEs-0.012SS, but the pore throat size of PHIPEs-0.015SS was smaller than those of PHIPEs-0.012SS. This was possibly because the internal phase volume fraction slightly decreased by raising the monomer amount of SS in the aqueous phase, and the smaller droplets were formed during the polymerization process. Moreover, it could be clearly seen that the surfaces of PHIPEs-0.012SS and PHIPEs-0.015SS were smooth, while the surface of PHIPEs-0.012SS-Ag and PHIPEs-0.015SS-Ag were rough, suggesting that the silver particles were immobilized on the cavity surface of the macroporous adsorbents. In addition, after the reaction between Ag^+^ and NaBH_4_, PHIPEs-SS-Ag retained the foam structure of PHIPEs-SS, indicating that this mild reaction condition had no negative effect on the porous morphology of the material. When compared with PHIPEs-0.015SS-Ag, the inferior mechanical characterization of PHIPEs-0.012SS-Ag was also observed, and this frangible nature could be attributed to the damaged polymer walls. Thus, PHIPEs-0.015SS-Ag was selected as the research object, namely PHIPEs-SS-Ag for subsequent studies.

**Fig. 3:**
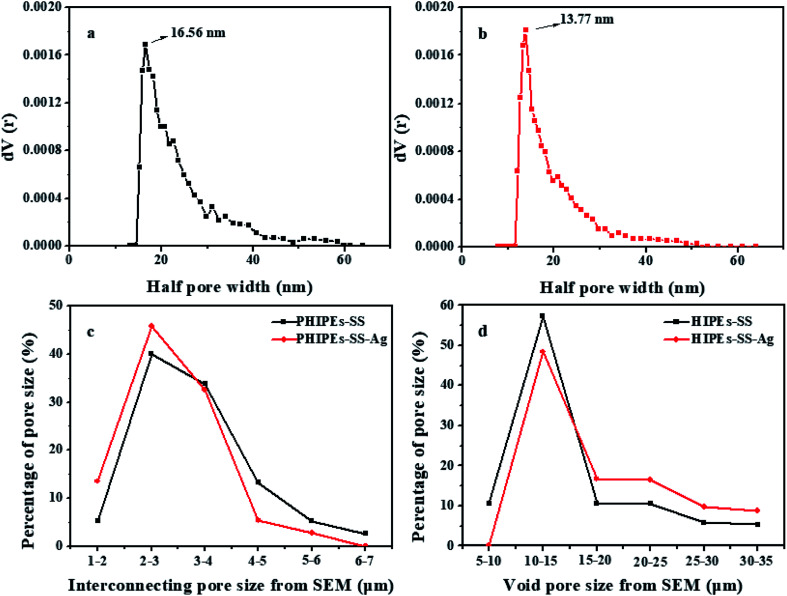
Half pore size distribution curves in the N_2_-sorption isotherm of PHIPEs-SS (a) and PHIPEs-SS-Ag (b); interconnecting pore size from SEM (c) and void pore size from SEM (d).

Fig. 3 shows the half pore size distribution of PHIPEs-SS (a) and PHIPEs-SS-Ag (b) from the N_2_ adsorption/desorption isotherms as well as the interconnecting pore size (c) and void pore size (d) from the SEM images.^36^ The surface area of PHIPEs-SS and PHIPEs-SS-Ag as determined from the Brunauer–Emmett–Teller (BET) adsorption method were 15.52 m^2^ g^−1^ and 16.55 m^2^ g^−1^. The slight increase in the surface area arose from the stacking of immobilized Ag nanoparticles. As shown in Fig. 3a and b, there were no obvious differences in the half pore size distribution of PHIPEs-SS and PHIPEs-SS-Ag; however, the average half pore size distribution of PHIPEs-SS-Ag slightly decreased from 16.56 nm to 13.77 nm. This was possibly because few Ag nanoparticles blocked the pores of the foam structure. The interconnecting pore and void pore sizes of PHIPEs-SS nearly experienced no change, as determined from the SEM images, when compared with PHIPEs-SS-Ag (Fig. 3c and d).

**Fig. 4:**
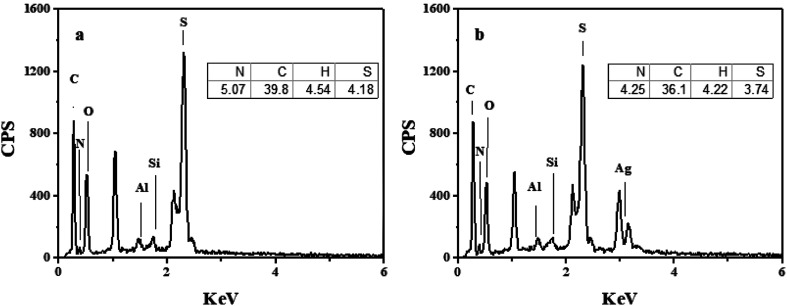
Energy dispersive spectrometry (EDS) and elemental analysis of PHIPEs-SS (a) and PHIPEs-SS-Ag (b).

The energy dispersive spectrometer (EDS) analysis for PHIPEs-SS and PHIPEs-SS-Ag were listed in Fig. 4a and 4b, respectively. As shown in Fig. 4a, the peaks of the elements Si and Al indicated that HNTs were attached as stabilized particles. Also, the peak of the element S was obviously displayed, which suggested that –SO_3_H groups from the SS monomer were also introduced *via* the polymerization reaction. When compared with Fig. 4a, the peak of the element Ag appeared in PHIPEs-SS-Ag (Fig. 4b), confirming the existence of Ag. The results of elemental analysis were also shown in the inset figures. The amount of S in PHIPEs-SS-Ag slightly decreased by 0.44% after the immobilization of the Ag particles. The results demonstrated the mild condition between immobilized Ag^+^ and NaBH_4_.

**Fig. 5:**
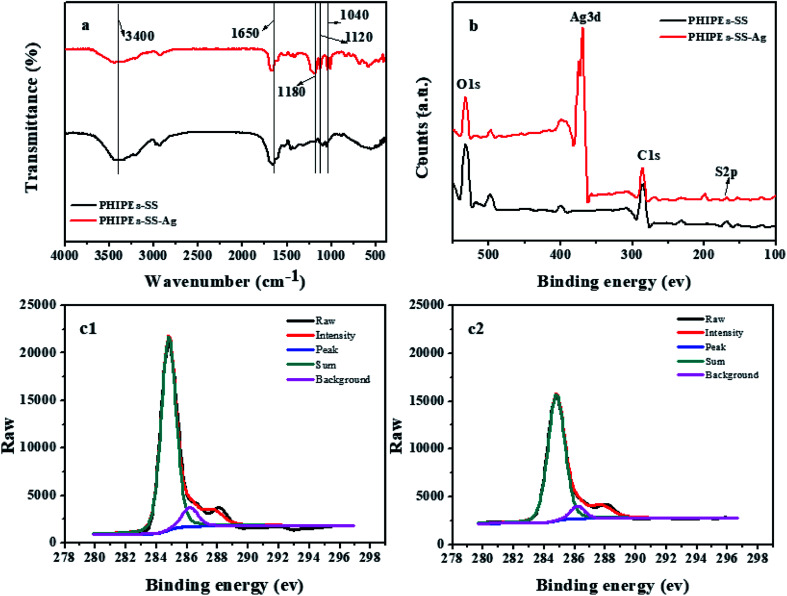
FT-IR spectra (a); X-ray photoelectron spectroscopy spectrums of survey (b); C1s of PHIPEs-SS (c1) and PHIPEs-SS-Ag (c2).

FT-IR spectra (a), the X-ray photoelectron spectroscopy spectrum of the survey (b), and the C1s of PHIPEs-SS (c1) and PHIPEs-SS-Ag (c2) were observed in Fig. 5. FT-IR analysis was carried out to verify the composition of PHIPEs-SS and PHIPEs-SS-Ag. The absorption bands at 3400 cm^−1^ and 1650 cm^−1^ corresponded to the –NH_2_ and C=O stretching vibrations from the AM monomer. The characteristic bands of 1440 cm^−1^ and 1120 cm^−1^ were the signals of the benzene ring and –SO_3_H groups from the SS monomer,^31,37^ respectively. The peak ascribed to the C–H bond was observed around 870 cm^−1^. The band around 1040 cm^−1^ associated with the O–H bending vibration, confirming the presence of HNTs. For PHIPEs-SS-Ag, an additional vibrational band around 1180 cm^−1^ could be assigned to the Ag, and this result was also confirmed by EDS analysis.

X-ray photoelectron spectroscopy (XPS) spectra of PHIPEs-SS and PHIPEs-SS-Ag were shown in Fig. 5b. The binding energies appearing at 531.83 eV, 284.79 eV, and 168.1 eV can be attributed to the O1s, C1s, and S2p electrons, respectively, suggesting the presence of –SO_3_H groups. Accordingly, the high-resolution XPS spectrum of C1s (Fig. 5c) showed signals around 284.6 eV, 286.2 eV and 287.3 eV, which were associated with C–C, C–SO_3_^−^ and O=C–N bonds,^38^ respectively. These results further revealed that the two monomers were involved in the polymerization reaction and that the –SO_3_H groups were successfully introduced into the network. Furthermore, a new peak located at 168.1 eV could only be found in PHIPEs-SS-Ag, demonstrating that the Ag nanoparticles were facilely attached to PHIPEs-SS.

**Fig. 6:**
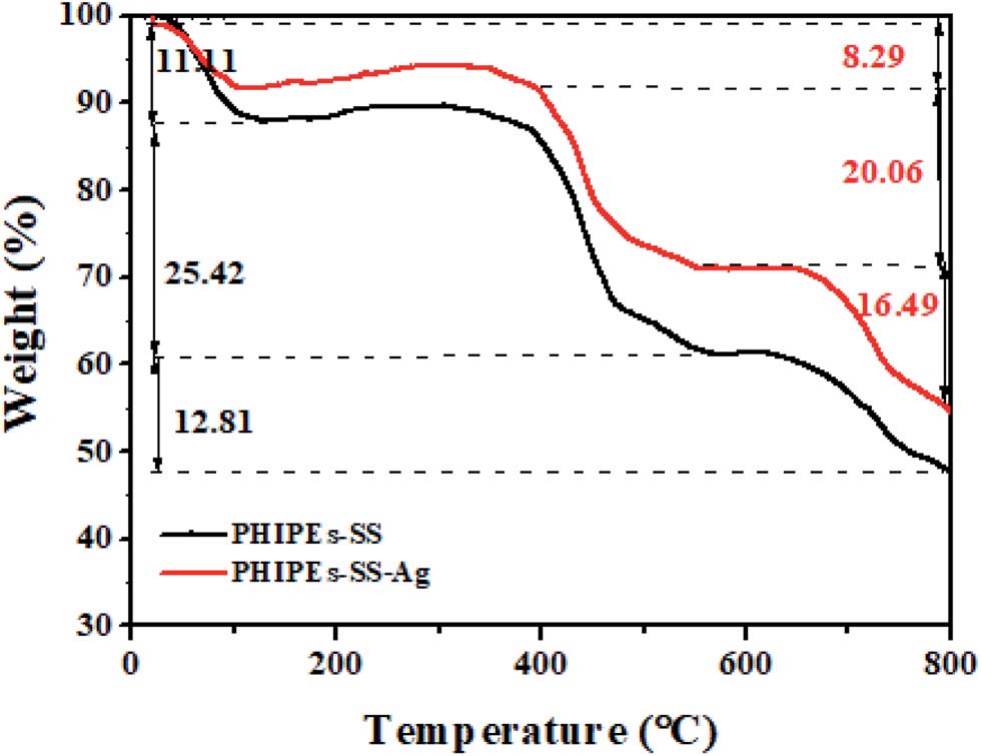
TGA curves of PHIPEs-SS and PHIPEs-SS-Ag.

Fig. 6 displayed the thermogravimetric (TGA) analysis of PHIPEs-SS and PHIPEs-SS-Ag. With the temperature increasing from 20 °C to 800 °C, there were three weight loss stages. In the first stage (before 100 °C), PHIPEs-SS and PHIPEs-SS-Ag both experienced a slight weight loss, which could be caused by the evaporation of water in the materials. During the second stage (390 °C to 550 °C), the weight loss of PHIPEs-SS and PHIPEs-SS-Ag were 25.42% and 20.06%, respectively, owing to the thermal decomposition of functional groups, such as the –SO_3_H groups. In the last stage (above 660 °C), the conspicuous weight losses may be ascribed to the calcination of the polymer network.

### 3.3 Effect of pH

**Fig. 7:**
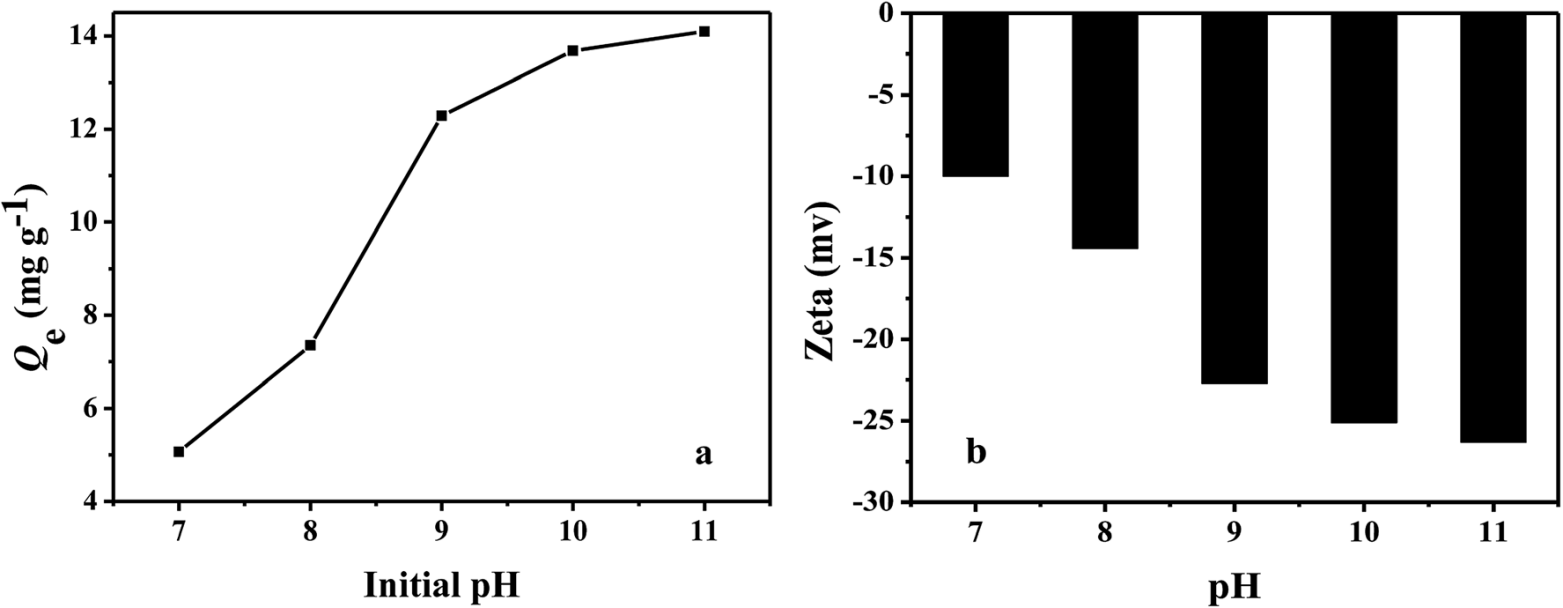
Effect of pH on PHIPEs-SS-Ag for the Li^+^ adsorption capacities at 25 °C (a) and the zeta potential of PHIPEs-SS-Ag at 25 °C (b).

The effect of pH on the adsorption capacities of PHIPEs-SS-Ag for Li^+^ at 25 °C was illustrated in Fig. 7a, and the initial concentrations of Li^+^ was fixed at 150 mg L^−1^. It was obviously observed that the adsorption capacity of Li^+^ increased along with the increase of solution pH,^39^ eventually reaching a constant value (at a pH ranging from 10 to 11). Additionally, the adsorption capacity attained a maximum value (*i.e.* 14.09 mg g^−1^) at pH = 11. The zeta potentials were listed in Fig. 7b. The results confirmed the negatively charged surface of PHIPEs-SS-Ag in the tested pH range, especially under strong alkali conditions. Thus, the adsorption mechanism between PHIPEs-SS-Ag and Li^+^ was electrostatic attraction. Moreover, to avoid possible environmental pollution and the hydrolysis of coexisting metal ions, pH = 10 was feasible for effective adsorption in our future work.

### 3.4 Adsorption kinetics

**Fig. 8:**
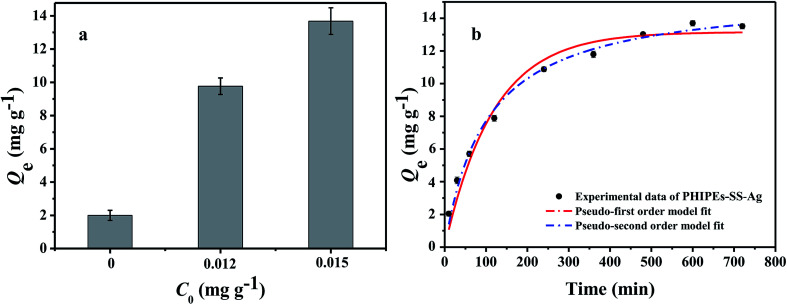
Adsorption amount for three kinds of PHIPEs-SS-Ag (a); kinetic data and modeling for the adsorption of Li^+^ onto PHIPEs-SS-Ag (b) (298 K, pH = 10, *C*_0_ = 150 mg L^−1^.)

It was evident that the adsorption amount of PHIPEs-0.015SS-Ag was higher than that of PHIPEs-0SS-Ag and PHIPEs-0.012SS-Ag; thus, PHIPEs-0.015SS-Ag (namely PHIPEs-SS-Ag in the following discussion) was selected for adsorption performance studies (Fig. 8a). The kinetic curves for Li^+^ adsorption onto PHIPEs-SS-Ag (pH = 10, 25 °C) was shown in Fig. 8b. As shown in Fig. 8b, the adsorption kinetics curve of PHIPEs-SS-Ag could be divided into two processes, a rapid binding during the initial 200 min and a slow process ranging from 200 min to 500 min. In the first stage of Li^+^ adsorption, the binding rate was fast and 80.59% of the equilibrium capacity was obtained due to the abundance of –SO_3_H groups. For the slow stage, the growth of the binding rate was not obvious because of insufficient adsorption sites, and the equilibrium time was observed after 300 min. Moreover, it could be clearly observed that the kinetic curves were continuous and smooth, suggesting the possible fact of the single layer coverage of Li^+^ onto the surface of PHIPEs-SS-Ag.^40^

In order to further study the adsorption kinetics of Li^+^ onto the PHIPEs-SS-Ag surface, the pseudo-first-order kinetic model (PFOKM) and pseudo-second-order kinetic model (PSOKM) were employed to analyze the kinetic results.^41^ The PFOKM and PSOKM were based on the following two equations, which are listed as eqn (2) and (3), respectively:

2 *Q*_*t*_ = *Q*_e_ − *Q*_e_e^−*k*_1_*t*^

3
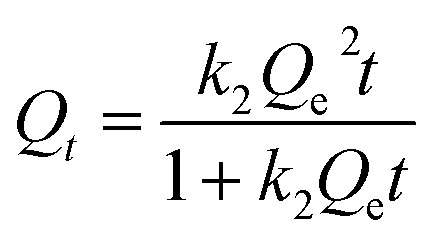
where *Q*_*t*_ (mg g^−1^) and *Q*_e_ (mg g^−1^) are the adsorption amounts of Li^+^ by PHIPEs-SS-Ag at time *t* and at equilibrium, respectively, and *k*_1_ (min^−1^) and *k*_2_ (g mg^−1^ min^−1^) are the rate constants from the pseudo-first-order and pseudo-second-order models, respectively.

The modeling for the adsorption of Li^+^ onto PHIPEs-SS-Ag was shown in Fig. 8b, and the adsorption rate constants and linear regression values were summarized in Table 1. The obtained adsorption amount from PSOKM was closer to the experimental data, and the *R*^2^ value from the two equations also confirmed that Li^+^ adsorption could be well described by PSOKM. This fact could also be proven by the results in Fig. 8b.

**Table 1:** Adsorption rate constants and linear regression values from two kinetic equations

*Q*_e,e_ a (mg g^−1^)	Pseudo-first-order kinetic model			Pseudo-second-order kinetic model		
	*Q*_e,c_ b (mg g^−1^)	*k*_1_ (min^−1^)	*R* ^2^	*Q*_e,c_ (mg g^−1^)	*k*_2_ (g mg^−1^ min^−1^)	*R* ^2^
13.682	6.5792	8.52 × 10^−3^	0.9696	7.7627	1.27 × 10^−3^	0.9902

a *Q*_e,e_ (mg g^−1^) is the experimental value of *Q*_e_.

b *Q*_e,c_ (mg g^−1^) is the calculated value of *Q*_e_.

### 3.5 Adsorption isotherm

**Fig. 9:**
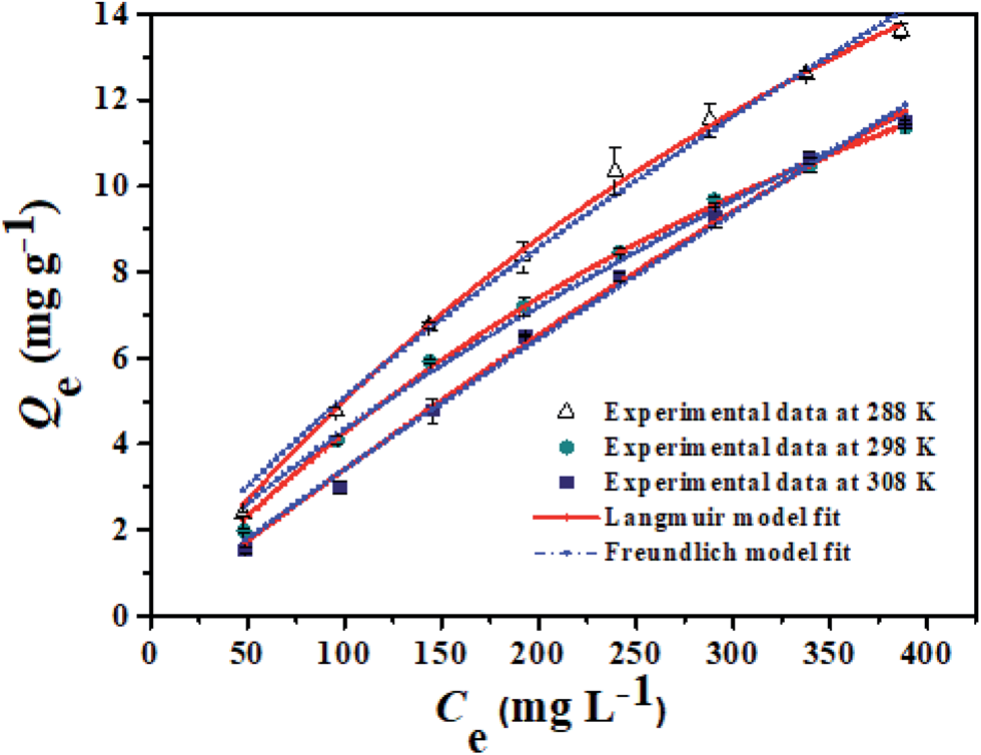
Equilibrium data and modeling for the adsorption of Li^+^ onto PHIPEs-SS-Ag at 288 K, 298 K, and 308 K, respectively.

In order to obtain information about the adsorption capacity, it is necessary to study the adsorption equilibrium. Thus, the adsorption equilibrium performance of PHIPEs-SS-Ag in the single system was evaluated by equilibrium adsorption experiments at three different temperatures. Moreover, Fig. 9 displayed two isotherm models by fitting to the adsorption equilibrium data of PHIPEs-SS-Ag, which could be acquired from the Langmuir^42^ and Freundlich^43^ simulant curves. Moreover, the calculated values of *R*^2^ and the adsorption equilibrium constants were shown in Table 2. Two classic isotherm models are expressed according to the following nonlinear equations:4
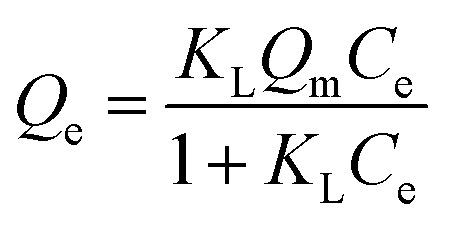

5 *Q*_e_ = *K*_F_*C*^1/*n*^_e_

where *Q*_e_ (mg g^−1^) is the value of Li^+^ adsorbed at equilibrium, *Q*_m_ (mg g^−1^) is the maximum adsorption capacity of PHIPEs-SS-Ag, *C*_e_ (mg L^−1^) is the equilibrium concentration of Li^+^, *K*_F_ (mg g^−1^) is the Freundlich adsorption equilibrium constant, and *K*_L_ (L mg^−1^) is the Langmuir adsorption equilibrium constant.

**Table 2:** Adsorption equilibrium constants from Langmuir and Freundlich isotherm equations

*T* (*K*)	Langmuir isotherm model			Freundlich isotherm model		
	*R* ^2^	*Q*_m,L_ (mg g^−1^)	*k*_L_ (L mg^−1^)	*R* ^2^	*K*_F_ (mg g^−1^)	1/*n*
288	0.9943	59.85	6.25 × 10^−4^	0.9912	0.0583	0.8911
298	0.9977	35.06	1.67 × 10^−2^	0.9905	0.1608	0.7503
308	0.9982	27.09	1.90 × 10^−2^	0.9900	0.1509	0.7294

From the results of PHIPEs-SS-Ag at three different temperatures (Fig. 9), it was found that the adsorption capacity decreased with increasing temperature. The equilibrium adsorption capacity of PHIPEs-SS-Ag for Li^+^ at 288 K was 59.85 mg g^−1^, which was larger than the values at 298 K and 308 K. Moreover, it was also observed that the Langmuir isotherm model can better represent the adsorption equilibrium behavior of Li^+^ onto PHIPEs-SS-Ag than the Freundlich model by comparing the values of *R*^2^ in Table 2, indicating the binding nature of the monolayer adsorption reactions. 1/*n* is a value used to measure the exchange intensity or surface heterogeneity, which can describe the removal condition. In this study, the values of 1/*n* for PHIPEs-SS-Ag at three different temperatures (288 K, 298 K, 308 K) were 0.8911, 0.7503 and 0.7294, respectively, which illustrated that the capture condition of PHIPEs-SS-Ag was more beneficial at a lower temperature.

### 3.6 Adsorption selectivity, reusability and antibacterial capability

**Fig. 10:**
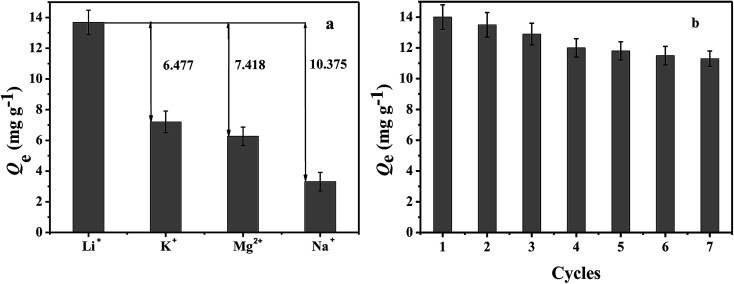
Adsorption capacity of PHIPEs-SS-Ag for Li^+^, K^+^, Mg^2+^, and Na^+^ (a). Adsorption–regeneration capacity of PHIPEs-SS-Ag for Li^+^ removal (initial [Li^+^] = 150 mg L^−1^) (b).

**Fig. 11:**
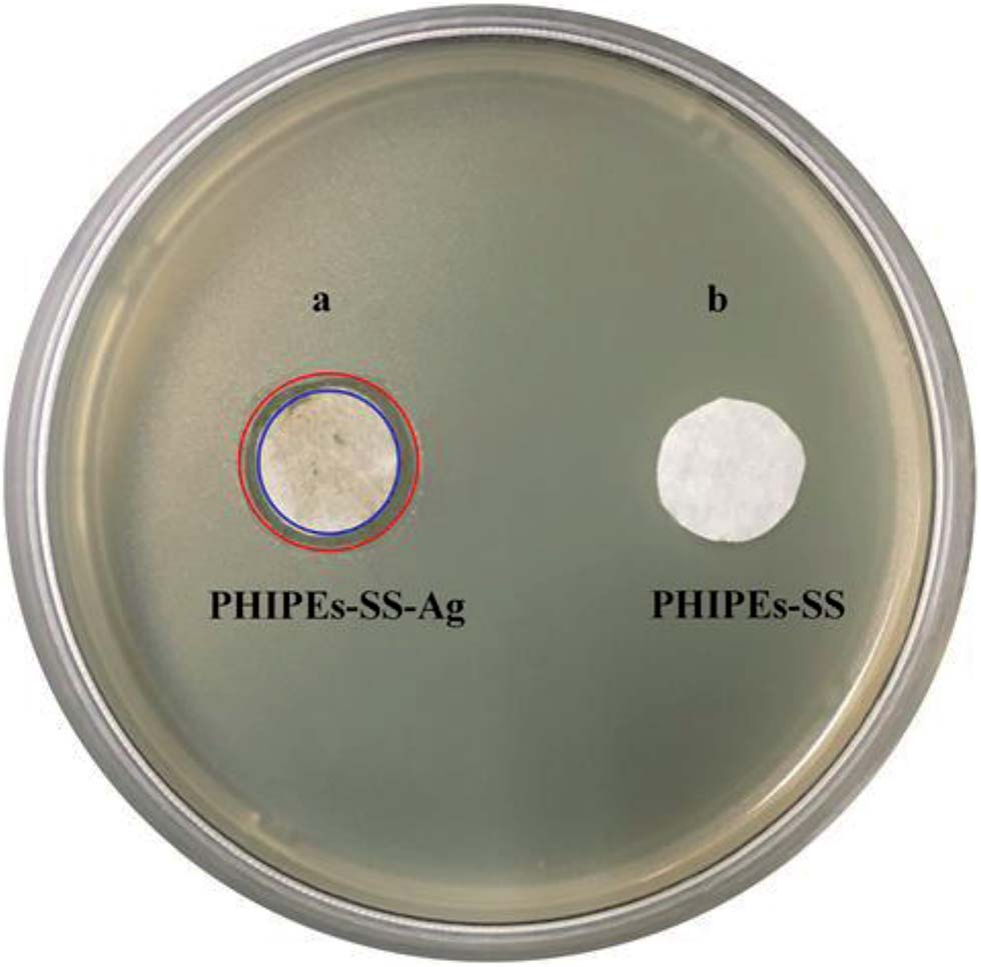
Inhibition zones of PHIPEs-SS-Ag (a) and PHIPEs-SS (b).

Adsorption selectivity is an extremely important factor used to evaluate the ability of an adsorbent. In this work, K^+^, Mg^2+^, and Na^+^ were considered as the competitive ions to study the adsorption selectivity of PHIPEs-SS-Ag for Li^+^. As shown in Fig. 10a, PHIPEs-SS-Ag possessed excellent adsorption ability for Li^+^, and the capture amount was superior to that of the other competitive ions. As shown in Fig. 10b, after seven sequential cycles of adsorption–regeneration, the adsorption capacity for Li^+^ remained at 80.71% of the initial value, owing to the reduction of binding sites in PHIPEs-SS-Ag. Thus, it could be concluded that PHIPEs-SS-Ag had good regeneration ability for the selective capture of Li^+^. The antimicrobial activity of PHIPEs-SS and PHIPEs-SS-Ag, detecting against the model *E. coli* bacterial strains by forming a zone of inhibition,^44^ suggested that PHIPEs-SS-Ag possessed antibacterial performance, which was beneficial for the antifouling process (Fig. 11).

## 4. Conclusions

In this work, an effective method was demonstrated to fabricate silver-modified porous polystyrene sulfonate (PHIPEs-SS-Ag) *via* the high internal phase emulsion template and post-synthetic modification. PHIPEs-SS-Ag possessed a highly porous foam structure, an abundance of adsorption sites (*i.e.*, sulfonic acid functional groups), and immobilized Ag nanoparticles. PHIPEs-SS-Ag exhibited an excellent adsorption amount, binding kinetics, selectivity, regeneration, and antifouling performance. Our proposed synthesis strategy can be facilely used to prepare functional adsorbents for the selective extraction of Li^+^.

## Conflicts of interest

There are no conflicts to declare.

## Supplementary Material
